# Kinetics, prognostic and predictive values of *ESR1* circulating mutations in metastatic breast cancer patients progressing on aromatase inhibitor

**DOI:** 10.18632/oncotarget.12950

**Published:** 2016-10-27

**Authors:** Florian Clatot, Anne Perdrix, Laetitia Augusto, Ludivine Beaussire, Julien Delacour, Céline Calbrix, David Sefrioui, Pierre-Julien Viailly, Michael Bubenheim, Cristian Moldovan, Cristina Alexandru, Isabelle Tennevet, Olivier Rigal, Cécile Guillemet, Marianne Leheurteur, Sophie Gouérant, Camille Petrau, Jean-Christophe Théry, Jean-Michel Picquenot, Corinne Veyret, Thierry Frébourg, Fabrice Jardin, Nasrin Sarafan-Vasseur, Frédéric Di Fiore

**Affiliations:** ^1^ Department of Medical Oncology, Henri Becquerel Centre, Rouen, France; ^2^ INSERM U918, Henri Becquerel Centre, Rouen, France; ^3^ EquIpe de Recherche en Oncologie, Rouen, France; ^4^ Department of Biopathology, Henri Becquerel Centre, Rouen, France; ^5^ INSERM U1079, Faculty of medecine, Rouen, France; ^6^ Department of Gastroenterology, Rouen University Hospital, Rouen, France; ^7^ Department of Biostatistics, Rouen University Hospital, Rouen, France

**Keywords:** ESR1 mutation, digital PCR, breast cancer, aromatase inhibitor, kinetics

## Abstract

**Purpose:**

To assess the prognostic and predictive value of circulating *ESR1* mutation and its kinetics before and after progression on aromatase inhibitor (AI) treatment.

**Patients and methods:**

*ESR1* circulating D538G and Y537S/N/C mutations were retrospectively analyzed by digital droplet PCR after first-line AI failure in patients treated consecutively from 2010 to 2012 for hormone receptor-positive metastatic breast cancer. Progression-free survival (PFS) and overall survival (OS) were analyzed according to circulating mutational status and subsequent lines of treatment. The kinetics of *ESR1* mutation before (3 and 6 months) and after (3 months) AI progression were determined in the available archive plasmas.

**Results:**

Circulating *ESR1* mutations were found at AI progression in 44/144 patients included (30.6%). Median follow-up from AI initiation was 40 months (range 4-94). The median OS was decreased in patients with circulating *ESR1* mutation than in patients without mutation (15.5 versus 23.8 months, *P*=0.0006). The median PFS was also significantly decreased in patients with *ESR1* mutation than in patients without mutation (5.9 vs 7 months, *P*=0.002). After AI failure, there was no difference in outcome for patients receiving chemotherapy (n = 58) versus non-AI endocrine therapy (n=51) in patients with and without *ESR1* mutation. *ESR1* circulating mutations were detectable in 75% of all cases before AI progression, whereas the kinetics 3 months after progression did not correlate with outcome.

**Conclusion:**

*ESR1* circulating mutations are independent risk factors for poor outcome after AI failure, and are frequently detectable before clinical progression. Interventional studies based on *ESR1* circulating status are warranted.

## INTRODUCTION

Endocrine therapy with either tamoxifen or aromatase inhibitors (AIs) is a key treatment in post-menopausal hormone receptor positive (HR+) metastatic breast cancer (MBC) which has a median time before progression of less than one year [1]. An emerging activating mutation in the estrogen receptor gene (*ESR1*), which leads to a ligand independent receptor activity, is one of the major molecular events involved in AI resistance [2, 3]. First reported in 1997 [4], these mutations have been recently described as (i) an acquired alteration, with a presence in primary tumour in less than 2% of cases [5, 6]; (ii) frequent, with a detection rate approximately 20% in metastases of HR+MBC patients progressing after endocrine therapy [7, 8]; and (iii) recurrent, with 4 hot-spot mutations (D538G, Y537S/N/C) contributing to 74% of all *ESR1* acquired mutations [5].

Since 2015, several studies have shown that *ESR1* mutation detection in circulating tumour DNA (ctDNA) was clinically relevant and correlated with mutational status on metastasis [9–11]. In this context, digital PCR based-methods [12, 13] appear to be a more simple and sensitive approach for *ESR1* detection in ctDNA than next-generation sequencing (NGS) techniques [13, 14]. In large retrospective cohorts, *ESR1* mutations were found specifically in HR+ MBC patients treated by AI and were highly predictive of a lack of sensitivity to subsequent AI exposure [9]. Recently, some studies have reported a worse outcome after progression on AI with detectable circulating *ESR1* mutations [9, 15, 16]. Nevertheless, the predictive value of circulating *ESR1* mutation at progression on AI and its potential use in daily practice remains unclear.

In light of these results, circulating *ESR1* mutation detection may be a surrogate marker of AI resistance. In this context, based on archive plasmas we evaluated the prognostic and predictive values of circulating *ESR1* mutation detection in HR+MBC patients and analyzed its kinetics before and after progression on AI.

## RESULTS

### Patients characteristics and follow-up

**Figure 1:**
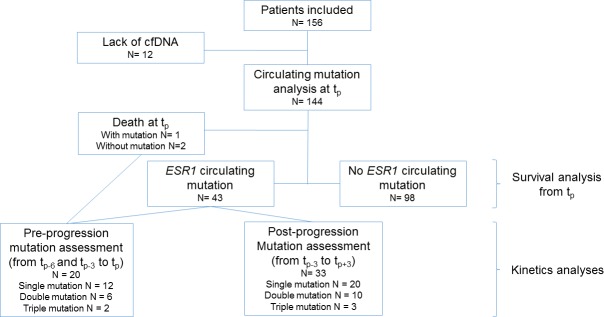
Diagram of the study

A total of 156 HR+MBC patients were included in this study. Due to the small amount of plasma DNA, the *ESR1* mutation status could not be performed for 12 patients. Therefore, the analysis was performed on 144 patients (Figure 1). The main characteristics of the population are summarized in Table 1.

**Table 1:** Baseline characteristics of patients with circulating ESR1 mutation versus patients without mutation

Characteristics	Wild type (*N*=100)	*ESR1* mutation (*N* = 44)	*P*
Median age at progression (years)	64.9	62.5	NS
HER2 status			
Positive	14(14)	3(7)	NS
Negative	84(84)	40(91)	
NC	2(2)	1(2)	
Disease presentation at metastatic setting*			
Denovo	46(46)	23(52)	NS
Relapsed	54(54)	21(48)	
Adjuvant treatment			
No	13(24)	3(14)	NS
Chemotherapy	30(56)	12(57)	
Homonotherapy	32(59)	17(81)	
Tamoxifen	26(48)	14(67)	
Aromatase inhibitor	12(22)	5(24)	
Ovarian suppression	5(9)	2(10)	
HER2 inhibitor	4(7)	1(5)	
Delay from adjuvant treatment			
During	9(16)	7(33)	NS
24 months	8(15)	2(10)	
NC	3(6)	1(5)	
Metastatic treatment before AI			
Total courses			
0	59(59)	31(70)	Ns
1	31(31)	8(18)	
2+	10(10)	5(11)	
Courses chemotherapy			
0	63(63)	31(70)	NS
1	29(29)	8(18)	
2+	8(8)	5(11)	
Courses endocrine therapy except AI			
0	92(92)	41(93)	NS
1+	8(8)	3(7)	
AI Introduction during metastatic disease			
Maintenance	26(26)	11(25)	NS
After progression	74(74)	33(75)	
AI treatment before progression			
AI exposure time			
<3 months	4(4)	0	0.002
3-6 months	25(25)	2(5)	
>6 months	71(71)	42(95)	
Median duration of exposure (months)	10.5	15	0.02
Treatment after progression on AI	7(7)	1(2)	NS
Switch to another AI	7(7)	1(2)	NS
Adding			
Everolimus	11(11)	3(7)	
HER2-inhibitor	2(2)	0	
Chemotherapy	39(39)	19(43)	
Tamoxifen/Fulvestrant	35(35)	16(36)	
No treatment/No modification of AI treatment	4(4)	3(7)	
Other treatment	2(2)	2(4)	

NOTE. Data are presented as No. (%) unless indicated otherwise

* Presentation of advanced disease is defined as denovo

(advanced at first presentation) or relapsed (relapsed after previous presentation with early stage cancer).

### *ESR1* mutational status at AI progression

At the time of AI progression (t_p_), at least one circulating *ESR1* mutation was detectable in 44 patients (30.6%). Overall, 63 mutations were found; D538G and Y537S were the two most frequent (24 and 21 samples, respectively), whereas Y537N and Y537C were detected in 16 and 2 samples, respectively. Among the 44 patients with mutation, 28 had a single circulating mutation, 13 had a double mutation and three had a triple mutation. The presence of a circulating *ESR1* mutation was not related to patient characteristics except for the median time of AI exposure, which was significantly longer in patients with *ESR1* mutations than in patients without mutation (15 *vs* 10.5 months, respectively, *P* = 0.02). The overall concordance between the 2 independent ddPCR analyses for each mutation was 98% (564 concordances over 576 analyses performed in duplicate).

### *ESR1* mutational status at AI progression and outcome

**Figure 2:**
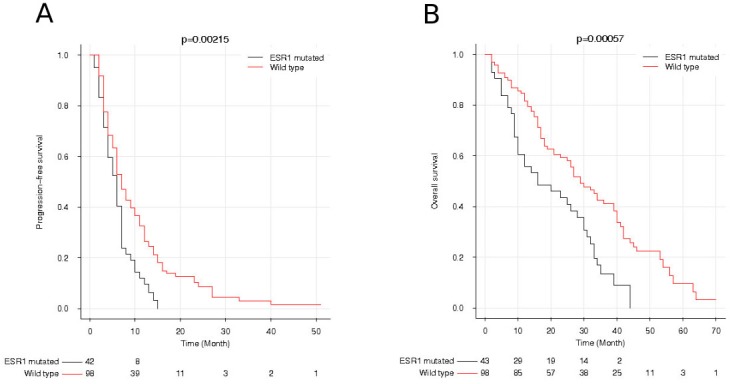
Progression-free survival (PFS) and overall survival (OS) after progression on first-line of aromatase inhibitor according to *ESR1* mutation status **A.** PFS of patients with or without *ESR1* mutation. **B.** OS of patients with or without *ESR1* mutation

The median time of follow-up from AI initiation to progression was 40 months (range 4-94). At time of analysis, 111 patients (77%) died and the 33 remaining were still under follow-up. Three patients (one mutated and two non-mutated) died at t_p_ and were excluded for the survival analysis. Among the 141 patients analyzed, the median overall survival (OS) was significantly lower in patients with circulating *ESR1* mutation (15.5 months, range 2-44) than in patients without mutation (23.8 months, range 2-70; *P* = 0.0006, HR = 1.9 CI [1.2-3.1], Figure 2B). The prognostic value of circulating *ESR1* mutation at AI progression was confirmed in multivariate analysis (*P* = 0.002, HR = 1.9 CI [1.3-3]). A WHO performance status > 1 (*P* < 0.0001, HR = 3 CI [1.9-4.7]); and a level of cell-free circulating DNA above the median value (HR = 1.8, *P* = 0.006 CI [1.2-2.7]) were also identified as independent prognostic factors of OS. A worse progression free survival (PFS) was observed in patients with *ESR1* mutation, with a median of 5.9 months, compared to 7.0 months for patients without mutation (*P* = 0.002, HR = 1.7 CI [1.1-2.5]). In the multivariate analysis of PFS, the presence of circulating *ESR1* mutation and a prior line of chemotherapy before AI introduction were identified as independent prognostic factors of worse outcome (*P* = 0.008, HR = 1.7 CI [1.2-2.5] and *P* = 0.009, HR = 2.3 CI [1.2-4.1], respectively) (Figure 2A).

### Predictive value for subsequent lines of treatment based on circulating *ESR1* mutations detection at AI progression

**Figure 3:**
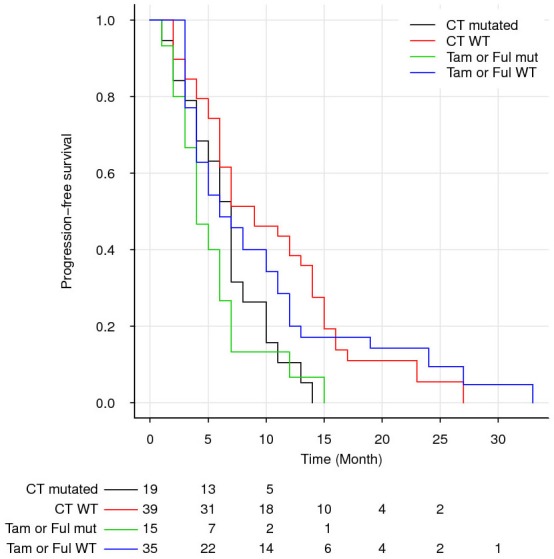
Overall survival (OS) after progression on first-line of aromatase inhibitor according to *ESR1* mutation status and post-progression treatment WT : wild-type. CT : chemotherapy. Tam: Tamoxifen. Ful : Fulvestrant.

After progression on AI, 51 patients received a non-AI endocrine therapy (34 tamoxifen and 17 fulvestrant), and 58 were treated with chemotherapy. The addition of everolimus or a Her2 inhibitor was used in only 14 and 2 patients, respectively. Finally, 8 patients were switched from one AI to another, 4 had no modification of AI treatment despite progression, 3 patients died at progression as previously mentioned and 4 patients received other treatments. The *ESR1* mutational status at AI progression did not selectively predict for better or worse outcome according to whether chemotherapy or non-AI endocrine therapy was given, since we observed similar PFS and OS between patients treated with chemotherapy-based regimens *versus* non-AI endocrine therapies in both mutated and non-mutated subgroups. Overall, patients with *ESR1* circulating mutation presented a worse OS than did patients without mutation regardless of treatment. Indeed, under chemotherapy, a median OS of 16 months was observed in case of mutation *versus* 27 months without mutation (*P* = 0.014, HR = 2.0 CI [1.01-4.0]). Similar results were observed in PFS: median PFS of 7 months in case of mutation *versus* 9 months without mutation (*P* = 0.009, HR = 1.9 CI [1.01-3.6]). When considering non-AI endocrine therapy, a trend over a worse survival in case of mutation was observed (median OS of 12 months in case of mutation *versus* 26 months without mutation, *P* = 0.09 HR = 1.7 CI [0.9-3.3] and median PFS of 5 months in case of mutation *versus* 6 months without mutation, *P* = 0.17, HR = 1.4 CI [0.8-2.8]) (Figure 3). When combining patients who received either chemotherapy or non-AI endocrine therapy after progression under AI, the median OS was significantly lower in patients with circulating *ESR1* mutation (16 months) than in patients without mutation (27 months; *P* = 0.002, HR = 1.9 CI [1.1-3.0]), confirming that the worse impact of circulating *ESR1* mutation observed over the whole population of the study was not related to patients receiving an AI-based treatment after progression.

Concerning the patients who were still treated by AI, or by AI plus everolimus, after progression under first-line of AI, the few number of patients analyzed and the heterogeneity of treatments received provide hardly interpretable data. Nevertheless, a trend over a better survival was observed in case of patients without mutation (median OS of 42 months without mutation *versus* 23 months with mutation, *P* = 0.17 HR = 2.1 CI [0.5-8.4]). In particular, the 11 non-mutated patients treated by AI+everolimus had a particularly long median OS of 46 months.

### Circulating *ESR1* mutation kinetics before progression

**Figure 4:**
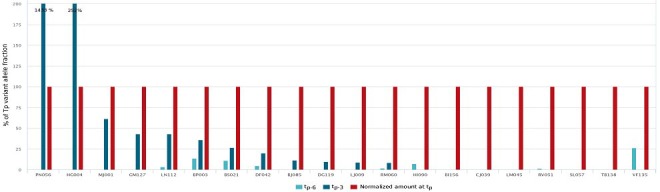
Pre-clinical detection of circulating ESR1 mutation This figure represents all the 20 patients for whom we had available plasmas at progression (t_p_), 3 months (t_p-3_) and 6 months(t_p-6_) before progression. Red color means a clinical progression at the time of mutational analysis whereas blue color means a stable or responding disease at the time of mutational analysis. To allow comparisons, the amount of circulating mutation have been normalized to 100 % for the time of progression. Circulating rates 3 (dark blue) or 6 (light blue) months before clinical progression are given in percentage of the value observed at progression.

Of the 44 patients presenting a circulating *ESR1* mutation at AI progression, plasma samples were available at 3 months before progression (t_p-3_) or 6 months before progression (t_p-6_) in 43 cases. In case of double or triple mutations, the predominant one was retained for subsequent analysis. For the 20 patients with samples at both t_p-3_ and at t_p-6_, 15 (75%) had detectable circulating *ESR1* mutations before AI progression, and in most of the cases, the amount increased over 3 months (*P* = 0.02, between t_p-6_ and t_p-3_ and *P* = 0.0087 between t_p-3_ and t_p_) (Figure 4). Of note, 2 patients had a decrease in the mutation amount between t_p-3_ and t_p_. For the 8 patients analyzed presenting double or triple mutations and with samples at both t_p-3_ and at t_p-6_, 5 (62%) patients had the predominant mutation detectable before t_p_ whereas non-predominant mutations were usually non-detectable (7/10 mutations) at both t_p-6_ and t_p-3_ or at a lower amount than the predominant one. For one patient, a Y537S mutation became predominant at t_p_ while the D538G mutation had a higher rate at both t_p-6_ and t_p-3_. For another patient, a decrease of a mutation contrasted with the appearance of another one suggesting a dissociated evolution of 2 sub-clones.

### Circulating *ESR1* mutation kinetics after progression

**Figure 5:**
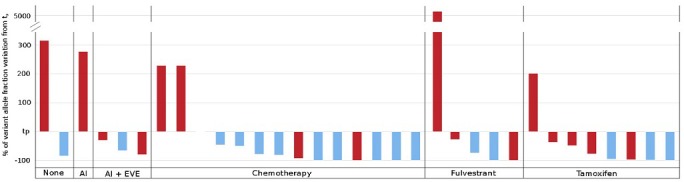
ESR1 circulating mutation evolution after progression and treatment change This figure represents all the 33 patients for whom we had plasma samples available 3 months after progression. The bottom of the figure indicates the post progression treatment received (AI: aromatase inhibitor, EVE: everolimus). The abscissa line is the normalized circulating *ESR1* mutation amount at time of progression. The bars represent the relative variation of this mutation amount 3 months after progression. The color of the bars is related to the clinical evolution observed 3 months after progression: blue color means stability or response and red color means a disease progression.

Circulating *ESR1* mutation kinetics after AI progression were analyzed in the 33/44 patients with mutation (75%) who had samples available at t_p+3_. Among them, 16 (48%) had a clinical progression, whereas the others had disease control or a response to the subsequent line of treatment. All patients (*n* = 6, 18%) presenting with an increase in circulating *ESR1* mutation at 3 months had disease progression. In contrast, among the remaining 27 patients presenting a decrease of circulating *ESR1* mutation, 10 (37%) experienced disease progression (Figure 5). In particular, 3 patients had no detectable mutation despite a clinical progression. For the 13 patients analyzed presenting double or triple mutation and with samples available at t_p+3_, only one patient (8%) had an increase of non-predominant mutations between t_p_ and t_p+3_ while the predominant mutation at t_p_ remained stable as well as the clinical evaluation at t_p+3_. For the 12 other patients, all the predominant and non-predominant mutations varied in the same direction between t_p_ and t_p+3_.

## DISCUSSION

Our results show that the detection of circulating *ESR1* mutation is relevant during AI exposure in HR+MBC patients. The present study, based on 144 patients, demonstrates the feasibility of *ESR1* detection in ctDNA and its clear association with AI resistance and a poor outcome for both PFS and OS. Furthermore, to our knowledge, we report for the first time that circulating *ESR1* mutations are detectable before clinical progression in approximatively 75% of patients. Taken together, our results highlight that *ESR1* mutation monitoring in ctDNA may help for decision making and early treatment change in MBC patients.

At progression, we observed an *ESR1* mutation frequency of 30.6%, which is consistent with those previously reported: 37% among 153 cases [11], 28.8% among 541 cases [16] and 25.3% among 395 patients [15]. In this study, *ESR1* mutations were also frequently polyclonal as previously reported [9, 10, 12, 14, 15, 17]. To note, the mutation frequency reported may be influenced by the number of *ESR1* mutations analyzed in each study, which usually vary from 2 [16] to 7 [15]. Therefore, the analysis in our study of 4 mutations may have underestimated the *ESR1* mutation frequency.

Our results showed that circulating *ESR1* mutation is associated with significantly worse outcomes with a difference of 8.3 months for OS and 1.1 months for PFS compared to patients without mutation. Similar results have been recently reported in a sub-group analysis of the BOLERO 2 trial (*n* = 541, OS of 32.1 months for patients without mutation *vs* 20.7 months for patients with mutation, *P* < 0.0001) [16], and in a sub-group analysis of the PALOMA-3 trial (*n* = 395, PFS of 9.2 months for patients without mutation *vs* 7.3 months in cases of mutation, *P* = 0.02) [15]. In contrast, Spoerke et al. did not find any difference related to *ESR1* mutation [11].

Surprisingly, in this study the median time of AI exposure during the metastatic setting was significantly longer in patients with *ESR1* mutations than in patients without mutation whereas it has not been reported previously. If confirmed, this result suggests that mechanisms for primary resistance to AI (progression in the first 3 or 6 months of AI exposure) may not be related to the emergence of an *ESR1* mutation. When considering the OS from the introduction of AI, a significantly worse outcome in case of circulating *ESR1* mutation was still observed (median OS of 37 months in case of mutation *versus* 47 without mutation, *P* = 0.027 HR 1.5 CI [0.99–2.4]).

In our study, post-AI progression treatment was mainly based on tamoxifen, fulvestrant or chemotherapy. To date, the addition of everolimus to AI [18] and, more recently, the addition of palbociclib to fulvestrant [19] has provided additional treatment options. Considering the sample size of patients analyzed for subsequent line, in our cohort, no particular treatment (chemotherapy, tamoxifen or fulvestrant) overcame the worse prognosis for patients with *ESR1* mutation. To note, this study was not randomized and therefore subject to biases in treatment allocation and was not powered enough for detecting a predictive value for each of the post progression treatment used. Concerning the addition of everolimus, Chandarlapaty et al. reported no PFS improvement for patients with the Y537S mutation but with a low number of cases in each arm; whereas wild-type patients or patients harbouring the D538G mutation had a better outcome when they were treated with AI + everolimus than with AI alone [16]. Concerning the potential use of palbociclib, a longer PFS for patients treated by fulvestrant + palbociclib compared to fulvestrant + placebo, both for *ESR1* wild-types and patients with mutation, has been reported [15]. In our study, no benefit of fulvestrant over tamoxifen was observed even if such a difference would be hardly detectable with the small sample sizes analyzed (*n* = 17 and *n* = 34, respectively). In contrast, the retrospective analysis of patients included in the randomized SoFEA study revealed that among the 63 patients with *ESR1* circulating mutation, a significantly better PFS was observed when a fulvestrant-based treatment was given, rather than exemestane (9.4 months *versus* 3.6 months, respectively, *P* = 0.002) [15]. Spoerke reported a similar outcome with the use of fulvestrant for patients with or without the *ESR1* mutation [11]. Moreover it has also been recently suggested that outcome after progression on AI treatment may be related to specific *ESR1* mutations [15]. Due to the numerous *ESR1* mutations, only large studies will be powered enough to answer this issue, which was not the case of our study. It is important to note that 26% of the mutations reported in the Spoerke et al. study were E380Q, for which the implication in the AI resistance has not been clearly established [20]. Thus, a dedicated analysis of mutations related to AI resistance, located on the codons 537 and 538, may have modified the survival analysis in this study. Moreover, pre-clinical data have shown that *ESR1* mutated cells show a partial resistance to fulvestrant in particular at clinical doses [2, 20]. More potent *ESR1* antagonists, such as AZD9496, have recently been reported to be more effective than fulvestrant (even used at supra-clinical doses) or tamoxifen in pre-clinical *ESR1* mutant breast tumour models [21]. Finally, unless not continuing single AI treatment, no definitive recommendation can be made on post-AI treatment based on the presence of a *ESR1* circulating mutation after progression on AI. Interestingly, the association of palbociclib to AI does not seem to prevent emergence of ESR1 mutations [22].

One of the strengths of our study was the availability of plasma samples at progression on AI, both before and after progression. Although we had previously reported the potential interest of *ESR1* mutation monitoring in plasma [12], the present study is the first, to our knowledge, that provides kinetics patients with *ESR1* mutation at t_p-6_, t_p-3_ and t_p_. Our results indicate that 75% of the mutations are already detectable when patients have not yet progressed. Even if this pre-clinical detection rate has to be confirmed by larger studies, it supports the potential interest of early treatment change based on the emergence of circulating mutation of resistance. The circulating mutation detection before clinical progression has already been reported after early breast treatment. In this study, 12 out of the 15 patients (80%) who relapsed had a detectable circulating mutation many months before clinical progression [23]. Nevertheless, this study was not restricted to HR+ tumors and used a patient-specific digital droplet PCR (ddPCR) assay designed after a massively parallel sequencing of the primary tumor. A restricted analysis of few recurrent mutations as performed in our study will be easier to transfer to daily practice. Concerning the increase in mutation amount observed in our study while patients are still receiving AI therapy, we can hypothesize that stopping AI before clinical progression may improve patient outcomes by interrupting the selection pressure. Nevertheless, this quantitative result must be interpreted cautiously because it was based on a retrospective analysis. We will need to collect prospective data of circulating mutation emergence, including analysis during progression and non-progression to determine the clinical relevance before an interventional study. Such a prospective study is currently ongoing in our centre (NCT02473120).

We also analyzed plasma samples at t_p+3_, while patients were receiving various treatments. In contrast to a homogeneous evolution of *ESR1* circulating kinetics before progression, after the end of AI exposure, we observed that *ESR1* mutation variation is more heterogeneous. Indeed, we found that an increase in the amount of mutation was related to progression for 100% of the patients, as also reported by Takeshita et al. (11 progressions in 13 patients) [10]. Due to the low number of patients analyzed, this value may be overestimated. In contrast, a decrease in the amount of mutation was related to various clinical outcomes. Similar results have recently been reported by Spoerke who suggested that all metastatic lesions may not harbour *ESR1* mutations and may have different responses to post-AI treatment [11]. Such molecular heterogeneity and the parallel genetic evolution of separate metastatic sites under treatment have already been demonstrated under a PIK3 inhibitor in breast cancer patients [24]. In this context, ctDNA has been reported as probably more accurate than circulating tumor cells (CTC) to explore tumor heterogeneity by blood analysis [25].

One of the key issues in the circulating mutation analysis is a rigorous definition of a positive threshold; however, there is no consensus regarding that definition. Wang et al. called a sample positive if: (a) the allele frequencies were > 0 after subtraction of background noise; (b) > 2 mutant droplets were repeatedly detected; and (c) allele frequency was > noise adjusted LOD for at least 3 independent assays [17]. For Schiavon et al., a mutation was considered positive with at least 2 *ESR1* mutant droplets [9]. Finally, Takeshita et al. focused on an increase in the amount of *ESR1* mutation between 2 samples rather than defining a positive threshold based on only one point [10]. In our opinion, mutation criteria must be reliable before potential clinical use.

In conclusion, the present study highlights the potential clinical value of using ddPCR to monitor circulating *ESR1* mutation during AI treatment for HR+MBC patients. We showed that the presence of circulating *ESR1* mutations is an independent factor for poor outcome in AI failure and that these mutations are also detectable before clinical progression. Although ddPCR provides quick and robust results, a standardized and validated definition of a mutated sample needs to be investigated before its potential use in daily practice. To improve the outcomes in patients with *ESR1* mutation, dedicated trials are also warranted, to test the potential benefit of an early treatment change in cases of circulating mutation emergence, and also to test specific treatments, such as *ESR1* modulators that are more potent than tamoxifen or fulvestrant, or the addition of palbociclib.

## PATIENTS AND METHODS

### Patients

All consecutive patients who were treated in our centre between 2010 and 2012 for HR+ MBC and who had clinical progression during first-line AI treatment were retrospectively included. Clinical progression was decided by our local Breast Tumor Board and based on either progression by RECIST criteria or symptomatic worsening despite radiographically stable disease. Previous non-AI treatment for metastatic disease was allowed. Patients who had previously been exposed to AI in an adjuvant setting were eligible if there was at least a two-year delay between the end of treatment and the diagnosis of metastatic relapse. Patients' clinical and histological baseline characteristics were collected, as were the outcomes of subsequent lines used after AI progression. In our centre, routine biological analyses are performed every 3 months for MBC patients, as well as CT-scan. Remaining plasmas after biological analyses are stored in our plasma bank. Therefore plasma samples were collected prospectively in consecutive HR+MBC patients, but the design of the study and the analyses were performed retrospectively. All patients signed a consent form allowing the conservation and study of their biological samples. The study was approved by the Institutional Review Board of the Henri Becquerel Center (registering order 1503B).

### Circulating *ESR1* status at AI progression and kinetics

All patients underwent blood sampling at the time of AI progression (t_p_) for *ESR1* mutation detection. In cases of detectable circulating *ESR1* mutation at t_p_, the kinetics of circulating mutations were analyzed in blood samples that were collected concomitant to the physical examinations 3 or 6 months before (t_p-3;_ t_p-6_) and after (t_p+3_) AI progression, respectively.

### Plasma DNA extraction

Blood samples were collected in heparinized tubes and processed within two hours after collection with one centrifugation at 2000 g (10 min) at 4°C before storage at 20°C. DNA was retrospectively extracted from 0.4 to 2 mL of stored heparinized plasma using a QIAamp^®^ Circulating Nucleic Acid Kit (Qiagen, Hilden, Germany). Double-stranded DNA quantification was performed by a fluorimetric method using a Quant-iT™ PicoGreen^®^ dsDNA Assay Kit (Invitrogen, Carlsbad, CA, USA) and a Twinkle LB970 microplate fluorometer (Berthold, Bad Wildbad, Germany).

### Droplet digital PCR (ddPCR) analysis

Droplet-based dPCR (ddPCR^TM^) platform (Qx200^®^ ddPCR System, Bio-rad Laboratories, Hercules, CA, USA) was used for detection of mutant circulating DNA in plasma samples. Four ng of circulating-free DNA (cfDNA) was preamplified as previously described [12]. Custom Taqman SNP genotyping assay (Life Technologies, Carlsbad, CA) for the detection of *ESR1* mutations Y537N, Y537S, Y537C and D538G (references and functional annotations of the *ESR1* mutations cited in that paper are available in the Supplementary Table 1). PCR cycling conditions and reagent compositions are described in Supplementary Table 2. Digital PCR conditions were optimized to identify the optimal annealing/extension temperature for each mutation, using wild-type DNA spiked with a mutant synthetic oligonucleotide inserted into pMT plasmid (Invitrogen, GeneArt^TM^,Carlsbad, CA, USA). Both for ddPCR assays optimization and samples analysis, the total copy number systematically comprised between 200 and 2000 copies/μL per reaction. In the case of an initial low copy number, 2 μL of heparinase (New England iolabs, Ipswich, MA, USA) was added to pre-amplification to reach the recommended copy number. At least 3 negative control wells with no DNA were included in every run. In case of too low amount of copy number despite use of heparinase, mutation detection could not be performed and the corresponding patient was excluded.

We serially diluted in triplicate *ESR1* mutant synthetic oligonucleotide into wild-type DNA to determine the ddPCR limit of detection (LOD), linearity and reproducibility (Supplementary Figure 1). Background noise is the minimum concentration of the mutant allele that can be differentiated from a negative control. To assess the background noise of our method, the allele burden was measured in 10 preamplified cfDNA extracted from healthy control heparinized plasma samples collected in the same conditions as the patient samples. Using the method of Hindson et al. [26], we defined the relevant threshold for each mutation to discriminate positive *versus* negative samples according to the reaction conditions (Supplementary Table 3). All data were analyzed using the QuantaSoft software (Bio-Rad, Hercules, CA, USA) and were manually reviewed to provide precise interpretation of data points. The variant allele fraction (VAF) was defined as the proportion of mutant DNA copies relative to the sum of mutant and wild-type DNA copies obtained by ddPCR. Samples at t_p_ were considered mutated if at least two independent ddPCR analyses by two independent operators found a VAF above the mutation threshold. ddPCR analyses were all performed blindly from clinical data. Samples at t_p-6_, t_p-3_ and t_p+3_ were analyzed on one or two runs according to the available samples.

### Statistical analysis

The primary endpoint was to evaluate the overall survival (OS) according to circulating *ESR1* mutation status at the time of AI progression. OS was calculated from t_p_ to the date of death from any cause. Blinded to the project outcome and considering that 30% of patients would have an *ESR1* mutation at progression on AI [27] and that 70% of the patients at progression on AI would die during the 3 following years [28], we determined that at least 121 patient records would have to be retrieved [29] to detect a hazard ratio of at least 2 between mutated and non-mutated *ESR1* patients with a power of 80% and an alpha of 5% using the Cox model to test group homogeneity. For secondary endpoints, we analyzed the frequency of circulating *ESR1* mutation at progression on AI, PFS and OS from t_p_ and under subsequent lines. Finally, we described the kinetics of the circulating *ESR1* mutation before and after AI progression. The prognostic factors of PFS and OS were determined in relation to the following variables: age, time delay from metastatic evolution to AI progression, WHO performance status at AI progression, cfDNA amount at progression, circulating *ESR1* mutation at AI progression, prior lines of treatment before AI introduction, duration of AI exposure, and type of treatment after AI progression. Comparisons between groups were made using the chi-squared test or the Wilcoxon test. Patients who died at t_p_ were excluded from survival analysis. Univariate analysis and survival curves were calculated using the Kaplan-Meier method and compared using the log-rank test. Multivariate analysis was performed using the Cox model and a backward method. All statistical analyses were performed using the R software version 3.0.1.

## SUPPLEMENTARY MATERIALS FIGURES AND TABLES



## Abbreviations

AI: Aromatase inhibitor
CI: Confidence interval
CTC: Circulating tumor cell
cfDNA: Circulating free DNA
ctDNA: Circulating tumor DNA
*ESR1*: Estrogen receptor gene
HR: Hazard Ratio
HR+: Hormone receptor positive
MBC: Metastatic breast cancer
NGS: Next generation sequencing
OS: Overall survival
PFS: Progression free survival
t_p_: time of AI progression
t_p-6_: 6 months before time of AI progression
t_p-3_: 3 months before time of AI progression
t_p+3_: 3 months after time of AI progression
VAF: Variant allele fraction
WHO: World Health Organization
