# Supplementary material for: Kinetics, prognostic and predictive values of ESR1 circulating mutations in metastatic breast cancer patients progressing on aromatase inhibitor
Source: Oncotarget. 2016 Oct 27;7(46):74448–59. doi: 10.18632/oncotarget.12950 (PMC5342678; doi:10.18632/oncotarget.12950)
Supplement: Supplementary file 1 [file oncotarget-07-74448-s001.pdf]

Kinetics, prognostic and predictive values of *ESR1* circulating mutations in metastatic breast cancer patients progressing on aromatase inhibitor

Supplementary Material

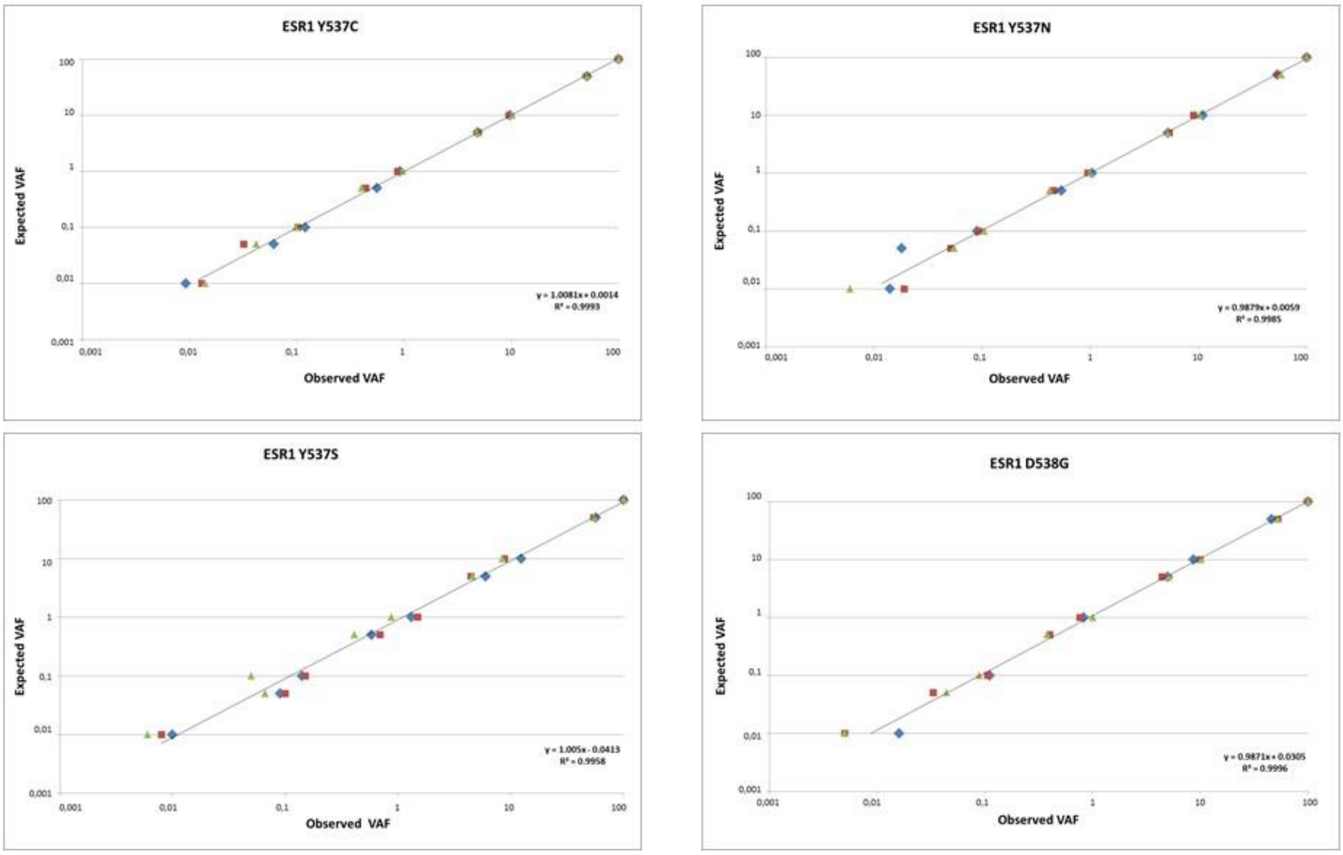

VAF : Variant allele fraction

Supplementary Figure 1

Determination of the limit of detection (LOD), linearity and reproducibility for each *ESR1* mutation ddPCR assay (analyses in triplicate)

Supplementary Table 1

References and functional annotations of the *ESR1* mutations cited

gene	mutation name	hg19 COSMIC reference	genomic coordinate	CDS mutation	AA mutation
ESR1	D538G	COSM94250	chr6:152419926	c.1613A>G	p.D538G
ESR1	Y537S	COSM1074639	chr6 :6:152419923	c.1610A>C	p.Y537S
ESR1	Y537N	COSM1074635	chr6:152419922	c.1609T>A	p.Y537N
ESR1	Y537C	COSM1074637	chr6:152419923	c.1610A>G	p.Y537C
ESR1	E380Q	COSM3829320	chr6:152332832	c.1138G>C	p.E380Q
ESR1	L536R	COSM4774826	chr6 :152419920	c.1607T>G	p.L536R
ESR1	S463P	COSM4771561	chr6 :152415537	c.1387T>C	p.S463P

Supplementary Table 2

ddPCR cycling conditions and reagent compositions

Step	PCR cycling	Number of cycles	Reagent composition
pre-amplification	95°C for 10 min	1	TaqMan universal PCR master mix, no UNG: 12.5 µL
	95°C for 15 sec<br>60°C for 4 min	12	Custom TaqMan dPCR Assay 20X: 0.25 µL
			DNA: 4ng<br>Heparinase: 2 µL
			Water, PCR grade: QSAD total volume: 25 µL
ddPCR Qx200	95°C for 10 min	1	Bio-Rad ddPCR Supermix for probes (No dUTP) 2X: 10µL
	94°C for 30 sec<br>54-56 °C for 1 min	40	Custom TaqMan dPCR Assay 20X: 1µL<br>cfDNA: 9µL of 10-fold diluted pre-amplified DNA; 9µL containing 25000 copies of plasmid DNA
	98°C for 10 min	1	Water, PCR grade, QSAD Total Volume 20µL

ESR1 mutation	ddPCR hybridation temperature
Y537S	54°C
Y537N	54°C
Y537C	56°C
D538G	55°C

Abbreviations: PCR, polymerase chain reaction; dPCR: digital PCR; DNA, deoxyribonucleic acid;

TE: 10 mM Tris-Cl and 1 mM EDTA (pH 8) buffer; cfDNA: circulating cell-free DNA. QSAD: Add quantity sufficient to make

Supplementary Table 3

Primers and probes sequences ; limit of detection values and thresholds of positivity for each mutation ddPCR assay

ESR1 Mutation	Primer and probe	Sequence
Y537S	Forward primer	5'-CTGTACAGCATGAAGTGCAAGAAC-3'
	Reverse primer	5'-GGCTAGTGGGCGCATGTAG-3'
	Wt probe	5'-TGCCCCTCTATGACCTGC-3'
	Mut probe	TGCCCCTCT**C**TGACCTGC
	LOD value	0.05%
	Threshold of positivity without heparinase	0.05%
	Threshold of positivity with heparinase	0.08%
Y537N	Forward primer	5'-CTGTACAGCATGAAGTGCAAGAAC-3'
	Reverse primer	5'-GGCTAGTGGGCGCATGTAG-3'
	Wt probe	5'-TGCCCCTCTATGACCTGC-3'
	Mut probe	5'TGCCCCTC**A**ATGACCTGC-3'
	LOD value	0.05%
	Threshold of positivity without heparinase	0.05%
	Threshold of positivity with heparinase	0.13%
D538G	Forward primer	5'-CTGTACAGCATGAAGTGCAAGAAC-3'
	Reverse primer	5'-GGCTAGTGGGCGCATGTAG-3'
	Wt probe	5'-TGCCCCTCTATGACCTGC-3'
	LOD value	0.05%
	Threshold of positivity without heparinase	0.25%
	Threshold of positivity with heparinase	0.42%
	Mut probe	5'-CCCTCTATG**G**CCTGCTGC-3'
Y537C	Forward primer	5'-CTGTACAGCATGAAGTGCAAGAAC-3'
	Reverse primer	5'-GGCTAGTGGGCGCATGTAG-3'
	Wt probe	5'-TGCCCCTCTATGACCTGC-3'
	Mut probe	5'-TGCCCCTCT**G**TGACCTGC-3'
	LOD value	0.05%
	Threshold of positivity without heparinase	0.36%
	Threshold of positivity with heparinase	0.47%

Wt : Wild type ; Mut: mutation
